# The Large Variability in Response to Future Climate and Land-Use Changes Among Large- and Medium-Sized Terrestrial Mammals in the Giant Panda Range

**DOI:** 10.3390/ani16030420

**Published:** 2026-01-29

**Authors:** Xuzhe Zhao, Junfeng Tang, Hongxia Xu, Huiliang Yu, Wei Wei, Zejun Zhang

**Affiliations:** 1 Hubei Key Laboratory of Biological Resources Protection and Utilization, Hubei Minzu University, Enshi 445000, China; xuzhe_zhao@126.com; 2 Administration of Shennongjia National Park, Shennongjia 442421, China; 13212767210@163.com (H.X.); snjpyuhl@163.com (H.Y.); 3 Key Laboratory of Southwest China Wildlife Resources Conservation, Ministry of Education, China West Normal University, Nanchong 637009, China; weidamon@163.com; 4 College of Pharmacy, Chengdu University of Traditional Chinese Medicine, Chengdu 611137, China

**Keywords:** climate change, land-use change, range shifts, modulating effect, mammals

## Abstract

While climate change and land-use change are known to interact in their effects on species’ range shifts, most studies have only focused on their isolated and combined effects, hindering a comprehensive understanding of their effects on future species distributions. In this study, we assessed the isolated and combined effects of future climate change and land-use change on range shifts of 23 large- and medium-sized terrestrial mammals in the giant panda (*Ailuropoda melanoleuca*) range, as well as the impact of land-use change on climate change effects, by comparing projections for three model configurations: (1) dynamic climate and static land-use covariates, (2) static climate and dynamic land-use covariates, and (3) dynamic climate and dynamic land-use covariates. We find that all three types of models projected an average net loss of suitable habitat areas across the 23 mammalian species. Moreover, we find that although there is a large variation in individual species’ responses to both the single climate change and single land-use change, climate change impacts dominate responses for most species, while land-use change impacts were nonsignificant or weak. Finally, the effect of land-use change on climate change projections reveals that land-use change could amplify or offset some of the negative effects of future climate change. These findings highlight that adopting effective land-based conservation management strategies to mitigate the impacts of future climate change on large- and medium-sized terrestrial mammals in the giant panda range is of great urgency and significance, but these strategies should take into account the large variation in individual species’ responses to both climate change and land-use change.

## Simple Summary

Understanding and predicting the effects of climate change and land-use change on the range shifts of species is crucial for biodiversity conservation. In this study, we assessed the isolated and combined climate change and land-use change effects, as well as the modulating effect of land-use change within climate scenarios, using a more realistic land-use dataset. We found that there is large variability in the responses of the 23 large- and medium-sized terrestrial mammals in the giant panda range to single or combined effects of future climate and land-use change, as well as the effect of land-use change on climate change projections. These findings should inform more useful dialogue determining the effect of climate change and land-use change on the future distribution of the 23 large- and medium-sized terrestrial mammals in the giant panda range and consequently guide future effective land-based conservation planning.

## 1. Introduction

Global climate change and human-induced land-use change during the Anthropocene have already led to unprecedented biodiversity loss worldwide [1,2]. Due to increasingly intensified climate change and land-use change, recent scientific consensus suggests that such trends will continue or even amplify in the future [3]. In this context, projecting how species will respond to future climate change and land-use change has become an important issue in ecology and conservation biology [4]. This is largely because such projections can provide useful dialogue informing conservation strategies to offset the negative effects of climate change and land-use change on biodiversity [5,6]. For example, projecting changes in the area of suitable habitat can contribute to identifying the potential priority conservation species that will be most likely to be at risk regarding the impacts of climate change and land-use change, which is also critical to avoid the future loss of biodiversity [2,7].

Currently, to make effective recommendations for conservation policy and management, many studies have projected future species distribution under the following three types of climate change and land-use scenarios: (i) ‘dynamic’ (i.e., change with time periods of projection) climate and ‘static’ (i.e., remain unchanged) land use, (ii) static climate and dynamic land use, and (iii) dynamic climate and dynamic land use [8,9,10]. One of the main reasons is that this model framework can help to distinguish the isolated and combined effects of climate change and land-use change on species’ range shifts [8]. Nevertheless, these studies may not accurately capture the climate change and land-use change effects on species distribution. On one hand, the land-use datasets used in these studies (e.g., the Finer Resolution Observation and Monitoring-Global Land Cover datasets; [11]) may not fully represent the future species’ habitat suitability [12], as these datasets either ignore the relatively static land-use patterns inside protected areas or take this important feature of protected areas into account but do not fully cover all protected areas [13]. Accordingly, the corresponding projections might be unrealistic, and the feasibility of the corresponding recommendations for conservation strategies might be problematic [14]. Therefore, forecasting models using the land-use datasets with static land-use patterns inside protected areas and dynamic land-use patterns outside protected areas may be superior in projecting future species distributions. However, very few studies have looked into the effect of such hybrid land-use change scenarios on the magnitude and direction of distributional changes of species.

On the other hand, recent studies have suggested that, in addition to the isolated and combined effects, assessing the modulating effect of land-use change within climate scenarios can improve our understanding of how species may respond to future environmental changes and can also contribute to making more effective recommendations for conservation policy and management. Actually, human-induced land-use patterns can amplify or buffer the impacts of climate change on species’ range shifts [15,16]. For example, human-induced habitat fragmentation may reduce the ability of species to adjust their distribution ranges, thereby further exacerbating the negative effects of climate change on the species [17]. On the contrary, habitat heterogeneity may provide a wider bioclimatic envelope for species, thus mitigating the negative effects of climate change [18]. Therefore, a better understanding of how climatic and land-use changes will interact to influence future species distributions is essential to inform effective conservation strategies, as climatic factors are less artificially controllable, but land-use patterns are subject to the more local control of managers and decision-makers [14]. Yet, to date, very few studies have assessed the modulating effect of land-use change within climate scenarios on the future distribution of species.

To fill these gaps, in this study we report a comprehensive evaluation of the effect of climate change and land-use change on the future distribution changes of large- and medium-sized terrestrial mammals in the giant panda range [19,20]. This region is located in a global biodiversity hotspot and therefore harbours a lot of rare and endangered large- and medium-sized terrestrial mammals, which makes this region immensely suitable for assessing species’ range shifts under future climate change and land-use change. Our specific objectives are (1) to quantify the range shifts of each species due to single and combined climate change and land-use change effects and (2) to assess the impact of land-use change on the climate change effects on species’ range shifts. According to our knowledge, our study is the first attempt to investigate the isolated and combined effects of climate and land-use change on the future distribution of the giant pandas, as well as the impact of land-use change on climate change effects, which would have important implications for species conservation.

## 2. Materials and Methods

### 2.1. Study Area and Species Occurrence Data

This study was conducted across the giant panda range where large- and medium-sized terrestrial mammals have been censused and reported [21,22]. We focused on Sichuan province, China, for 75% of the giant panda population are distributed there [19,20]. The giant panda range in Sichuan province, China, spreads across five mountains, including the Minshan, Qionglai, Daxiangling, Xiaoxiangling, and Lianshan mountains. The occurrence records for the large- and medium-sized terrestrial mammals in these five mountains were obtained from previously published datasets [22]. These datasets contain 13,630 occurrence records for 23 large- and medium-sized terrestrial mammals, with the original species occurrence data collected from the Fourth National Giant Panda Survey [20] and other data sources. For modelling species distribution and for consistency with our climate and land-use data (see below), we filtered and retained only one occurrence record within each 1 km × 1 km grid cell for each species. Finally, 11,228 occurrence records for all 23 large- and medium-sized terrestrial mammals were retained in the subsequent analysis (Table 1).

### 2.2. Current and Future Climate and Land-Use Variables

We used five bioclimatic variables and five land-use variables at ~1 km spatial resolution to model the current and future distributions of the 23 large- and medium-sized terrestrial mammals: mean annual temperature (BIO1), temperature seasonality (BIO4), temperature annual range (BIO7), precipitation of the driest month (BIO14), precipitation seasonality (BIO15), and the proportion of area covered by cropland (CL), forest (FL), shrubland (SL), urban green spaces (UGSL), and water bodies (WL) in grid cells. This is because these ten environmental variables are biologically relevant to large- and medium-sized terrestrial mammals and also have low multicollinearity with the absolute value of Pearson’s correlation coefficients < 0.7.

All the climate variables were obtained from the WorldClim dataset [23]. For the current period, the five climate variables are the average for the period 1970–2000, while for the future period, the five climate variables are the average for two periods, 2041–2060 (2050s) and 2061–2080 (2050s), under three representative concentration pathways (RCP2.6, RCP4.5, and RCP8.5), respectively. In particular, these future bioclimatic variables were extracted from the global circulation model, MRI-ESM2-0, which has been recommended for use in China [21].

All the land-use variables were obtained from the FROM-GLC database [11]. For the current period, the five land-use variables are the proportion of the area covered by these five land-use types in each 1 km × 1 km grid cell for the year 2010. For the future period, the five land-use variables are the proportion of the area covered by these five land-use types in each 1 km × 1 km grid cell for the years 2050 and 2070 under three representative concentration pathways (RCP2.6, RCP4.5, and RCP8.5), respectively. To generate a more reasonable land-use dataset for the years 2050 and 2070, we further assume that the proportion of each land-use type in each grid cell inside protected areas is static and therefore is equal to the values in the current period.

### 2.3. Species Distribution Modelling

We adopted an extropy model (MaxEnt; [24]) to make projections of potential distributions for the 23 large- and medium-sized terrestrial mammals under both current and future climate and land-use conditions. For each species, default parameter settings for the MaxEnt model were used as they produce the optimal model [22]. We used the most commonly used metric, the area under the receiver operating characteristic curve (AUC), to assess the performance of each model through a bootstrap sampling technique repeated ten times, with random subsets of 70% of the dataset for model training and the remaining 30% for model test. We also used a standard output of MaxEnt, ‘permutation contribution’, to assess the relative contribution of each of the ten environmental variables to shaping the current distribution of each species.

The above trained model for each species was then projected to current and future climate and land-use conditions. In order to assess the isolated and combined effect of climate change and land-use change and the modulating effect of land-use change within climate scenarios on the future distribution of the 23 large- and medium-sized terrestrial mammals, following previous studies [8,9], we constructed three types of variable sets for each RCP scenario and for each time period: (i) dynamic climate and constant land-use variable sets (CLIM), which are used to assess the isolated effect of climate change; (ii) static climate and dynamic land-use variable sets (LU), which are used to assess the isolated effect of land-use change; and (iii) dynamic climate and land-use variable sets (COMB), which are used for assessing the combined climate change and land-use change effects and the impact of land-use change on climate change effects together with the CLIM sets.

Consequently, for each species, we generated 10 current (10 replicates) and 180 future (10 replicates × 3 variable sets × 3 RCP scenarios × 2 time periods) habitat suitability maps. Following Araujo and New [25], for each species, we used an ensemble approach to obtain a single habitat suitability map for the current period and for each future scenario. That is, for each species and each scenario, we calculated the weighted mean habitat suitability map of the 10 habitat suitability maps by using the 10 AUC values as the weighting coefficient. Finally, all these maps were converted into binary presence–absence maps by using the weighted mean threshold as the maximum sum of sensitivity and specificity [26].

### 2.4. Statistical Analysis

All analyses were conducted based on the above binary presence–absence habitat suitability maps for the 23 large- and medium-sized terrestrial mammals. To explore the single and combined effects of climate change and land-use change on the distribution of the 23 large- and medium-sized terrestrial mammals under different future scenarios, we calculated the relative changes in the area of suitable habitat (*RCSH*) for each species and for each of the three variable sets under each of the six different future scenarios (3 RCP scenarios × 2 time periods), using the following equation:

(1) $$RCSH\;=\;\frac{{Area}_{future}-{Area}_{current}}{{Area}_{current}}\times 100\%,$$

where *RCSH* is the relative change in suitable habitat area, and ${Area}_{current}$ and ${Area}_{future}$ are the projected areas of suitable habitat in current and future periods, respectively.

Moreover, to explore the effect of land-use change on the climate change effects on the range shifts of the 23 large- and medium-sized terrestrial mammals, we also compared the difference in RCSH between the COMB and CLIM models for each species under each future scenario.

## 3. Results

### 3.1. Model Performance and Variable Importance

**Figure 1:**
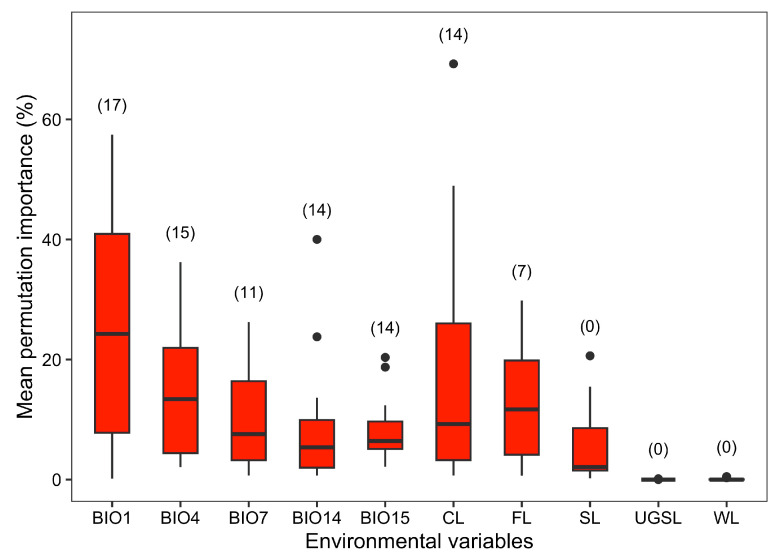
Mean permutation importance of the ten selected environmental variables in predicting the potential distributions of the 23 mammal species in the current period. BIO1: mean annual temperature, BIO4: temperature seasonality, BIO7: temperature annual range, BIO14: precipitation of the driest month, BIO15: precipitation seasonality; CL, FL, SL, UGSL, and WL are the proportion of the area covered by cropland, forest, shrubland, urban green spaces, and waters in the grid cells, respectively. The numbers in the brackets represent the number of species for which this variable was one of the four most important variables.

The mean training and test AUC values of our MaxEnt models for the 23 mammal species ranged from 0.771 to 0.941 and from 0.692 to 0.927, respectively, indicating that our models are sufficient to project the current and future distribution of all mammal species to some extent (Table 1). For the ten variables included in our models, mean annual temperature was included amongst the four most important variables for 17 of the 23 mammal species, followed by temperature seasonality (for 15 species), precipitation of the driest month (for 14 species), precipitation seasonality (for 14 species), proportion of farmland (for 14 species), temperature annual range (for 11 species), and proportion of area covered by forest (for 7 species), while the remaining three land-use variables (i.e., proportion of area covered by shrubland, urban green space, and water bodies) were not included amongst the four most important variables for any of the 23 mammalian species (Figure 1).

### 3.2. The Isolated Effect of Climate and Land Use on Habitat Suitability

**Figure 2:**
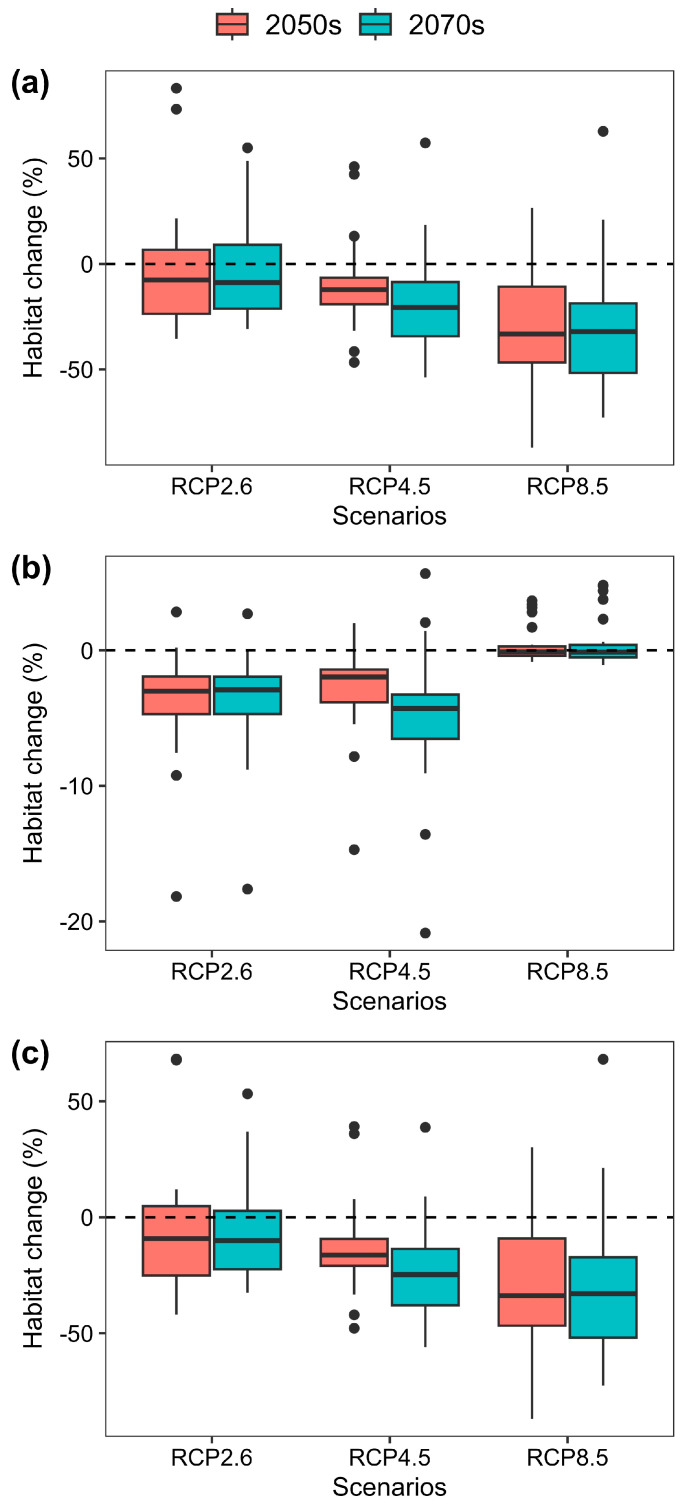
Habitat change (%) for the 23 mammal species projected by the (**a**) CLIM models (future climate and current land-use variables), (**b**) LU models (current climate and current land-use variables), and (**c**) COMB models (future climate and future land-use variables) by the 2050s and 2070s, respectively.

On average, the CLIM models projected an increase in net loss of suitable habitat for the 23 mammalian species from RCP2.6 to RCP8.5 and from the 2050s to the 2070s, respectively (Figure 2a). Specifically, the average changes in suitable habitat across the 23 mammalian species amounts to −1.5%, −9.5%, and −28.3% under RCPs 2.6, 4.5, and 8.5 by the 2050s and amounts to −2.3%, −17.7%, and −29.7% under RCPs 2.6, 4.5, and 8.5 by the 2070s, respectively. In addition, the individual species’ response under all climate change scenarios are also considerably variable. Specifically, the changes in the area of suitable habitat across the 23 mammalian species projected by the CLIM models range from −35.5% to 83.2%, from −46.7% to 46.1%, and from −87.1% to 26.6% under RCPs 2.6, 4.5, and 8.5 by the 2050s and from −30.8% to 55.0%, from −53.8% to 57.3%, and from −72.9% to 62.8% under RCPs 2.6, 4.5, and 8.5 by the 2070s, respectively.

In contrast, the LU models projected a net loss of suitable habitat for the 23 mammalian species from RCP2.6 to RCP8.5 and from the 2050s to the 2070s, respectively. However, the net loss of suitable habitat under RCP4.5 is less than under RCP2.6 by the 2070s, and it is less by the 2070s than by the 2050s under the RCP4.5 scenario (Figure 2b). Specifically, the predicted changes in suitable habitat area across all species on average amount to −4.0%, −3.0%, and 0.4% under RCPs 2.6, 4.5, and 8.5 by the 2050s and amount to −3.9%, −4.9%, and 0.6% under RCPs 2.6, 4.5, and 8.5 by the 2070s, respectively. Similarly, there is also large variability in the individual species’ response under all climate change scenarios, but the magnitude of the changes in the area of suitable habitats projected by the LU models for most species is less than the CLIM models (Figure 2a,b). Specifically, the changes in the area of suitable habitat across the 23 mammalian species projected by the LU models range from −18.2% to 2.8%, from −14.7% to 2.0%, and from −0.9% to 3.7% under RCPs 2.6, 4.5, and 8.5 by the 2050s and from −17.6% to 2.7%, from −20.9% to 5.7%, and from −1.1% to 4.8% under RCPs 2.6, 4.5, and 8.5 by the 2070s, respectively.

**Figure 3:**
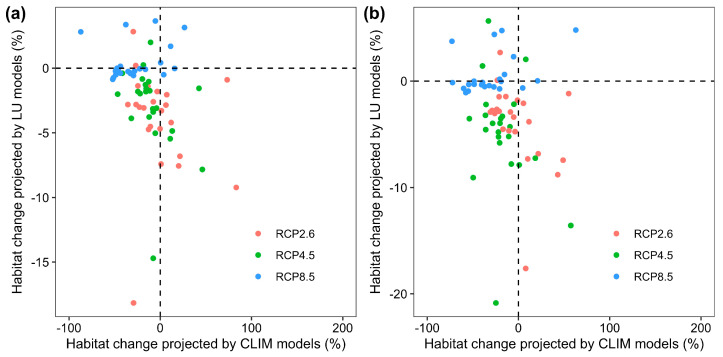
Relationship between predicted changes in suitable habitat area under future climate and land-use conditions (CLIM models) and current climate and future land-use conditions (LU models) for each species under all RCP scenarios by the 2050s (**a**) and the 2070s (**b**), respectively.

**Figure 4:**
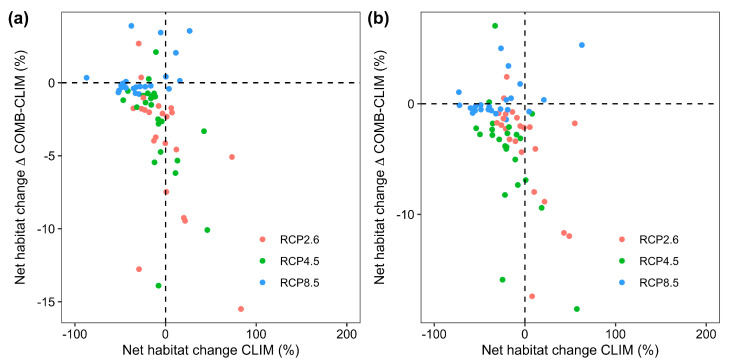
The impact of land-use change on climate change projections (i.e., the projected range shifts under future climate and land-use conditions (COMB models) minus the projected range shifts under future climate and current land-use conditions (CLIM models) for each species under all RCP scenarios by the 2050s (**a**) and the 2070s (**b**), respectively.

Moreover, by comparing the single effect of climate change and land-use change, there were also large variations in individual species’ response to the single effect of future climate change and land-use change across the 23 mammalian species (Figure 3). Specifically, the number of species that were predicted to experience (i) range expansion under both climate change and land-use change scenarios, (ii) range expansion under climate change scenarios but range contraction under land-use change scenarios, (iii) range contraction under climate change scenarios but range expansion under land-use change scenarios, and (iv) range contraction under both climate and land-use change scenarios across all RCP scenarios by the 2050s ranges from 0 to 3, from 2 to 9, from 2 to 4, and from 12 to 17, respectively, and ranges from 0 to 2, from 1 to 8, from 2 to 6, and from 13 to 17 by the 2070s, respectively (Figure 4).

### 3.3. The Modulating Effect of Land-Use Change Within Climate Scenarios

Similarly to the CLIM models, the COMB models on average also projected an increase in net loss of suitable habitat for the 23 mammalian species from RCP2.6 to RCP8.5 and from the 2050s to the 2070s, respectively (Figure 2c). Specifically, the predicted changes in suitable habitat across all species projected by the COMB models on average amount to −5.5%, −12.4%, and −27.9% under RCPs 2.6, 4.5, and 8.5 by the 2050s and amount to −6.1%, −22.2%, and −29.3% under RCPs 2.6, 4.5, and 8.5 by the 2070s, respectively. The COMB models also predicted larger variation in species’ responses than the LU models but smaller variation than the CLIM models. Specifically, the changes in the area of suitable habitat across the 23 mammalian species projected by the COMB models range from −42.0% to 68.2%, from −47.9% to 39.1%, and from −86.7% to 30.1% under RCPs 2.6, 4.5, and 8.5 by the 2050s and from −32.6% to 53.2%, from −56.1% to 38.8%, and from −72.4% to 68.1% under RCP2.6, RCP4.5, and RCP8.5 by the 2070s, respectively.

Moreover, the modulating effect of land-use change within climate scenarios (i.e., the difference in projected changes in the area of suitable habitat between the COMB and CLIM models) on species’ range shifts is also considerably variable (Figure 4). Specifically, 21, 21, and 15 species under RCP2.6, RCP 4.5, and RCP 8.5 by the 2050s and the 2070s, respectively, would lose additional suitable or gain less suitable habitat compared to the CLIM models, while for the remaining species, dynamic land-use change would offset habitat losses or promote habitat expansion from climate change.

### 3.4. Focus on One Representative and One Atypical Species

**Figure 5:**
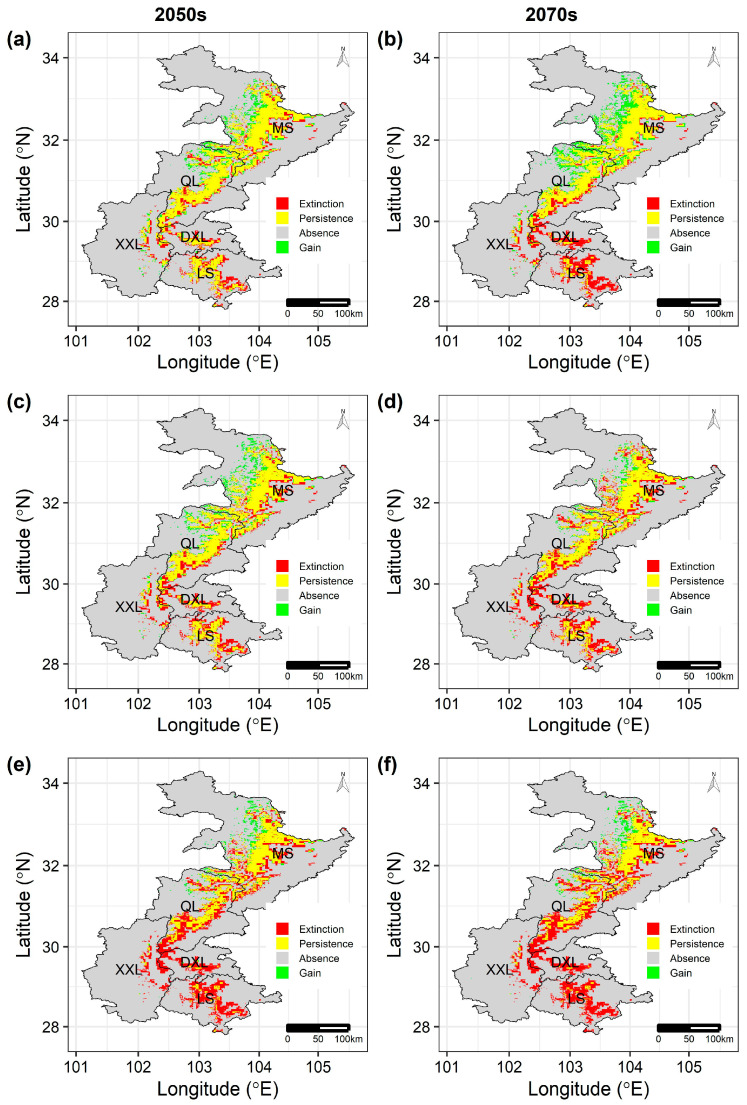
The predicted changes in suitable habitat of the giant panda between current and different future scenarios by the COMB models: (**a**) RCP2.6, (**c**) RCP2.6, and (**e**) RCP8.5 by the 2050s and (**b**) RCP2.6, (**d**) RCP2.6, and (**f**) RCP8.5 by the 2070s. Extinction: the grid cells that were predicted to be suitable in the current period but become unsuitable in future periods; Persistence: the grid cells that were predicted to be suitable in both current and future periods; Absence: the grid cells that were predicted to be unsuitable in both current and future periods; Gain: the grid cells that were predicted to be unsuitable in the current period but become suitable in future periods.

The giant panda was one of the numerous large- and medium-sized terrestrial mammals in our study area predicted to experience range contraction, and land-use change would lead to additional habitat loss of the giant panda under all future scenarios. Therefore, it is a representative species for most of the large- and medium-sized terrestrial mammals in our study area. Specifically, the predicted range contraction percentage was larger for the COMB models (14.4~54.8% under all future scenarios; Figure 5) than the CLIM models (13.5~54.5%; Figure S1) or LU models (0.2~2.2%; Figure S2). Moreover, like most of the mammals in this study, the giant panda was predicted to lose considerable suitable habitat in low latitudes, such as the Xiaoxiangling, Daxiangling, and Liangshan mountains, and gain some new suitable habitat in high latitudes, such as the Minshan Mountains (Figure 5, Figures S1 and S2).

**Figure 6:**
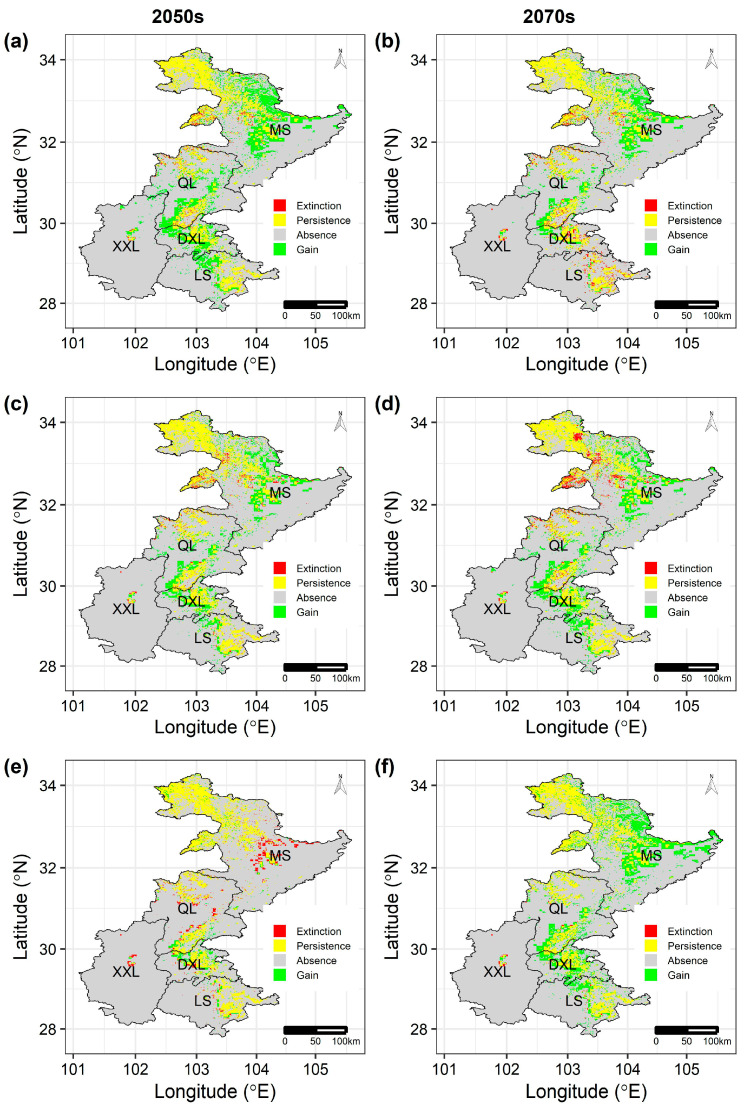
The predicted changes in suitable habitat of the rhesus macaque between current and different future scenarios by the COMB models: (**a**) RCP2.6, (**c**) RCP2.6, and (**e**) RCP8.5 by the 2050s and (**b**) RCP2.6, (**d**) RCP2.6, and (**f**) RCP8.5 by the 2070s. Extinction: the grid cells that were predicted to be suitable in the current period but become unsuitable in future periods; Persistence: the grid cells that were predicted to be suitable in both current and future periods; Absence: the grid cells that were predicted to be unsuitable in both current and future periods; Gain: the grid cells that were predicted to be unsuitable in the current period but become suitable in future periods.

On the contrary, the rhesus macaque was predicted to experience range expansion by both the CLIM and COMB models, but land-use change would lead to less habitat gains when comparing these two types of models, as the LU models predicted habitat loss of the rhesus macaque under all future scenarios expect the RCP8.5 scenario by the 2070s. Specifically, the predicted range expansion percentage was smaller for the COMB models (9.0~21.3% under all future scenarios; Figure 6) than the CLIM models (13.1~21.6%; Figure S3), while the range contraction percentage predicted by the LU models ranged from 0.02~7.2%; Figure S4. Moreover, the predicted habitat gains or losses were mainly located in the Minshan mountains (Figure 6, Figures S3 and S4).

## 4. Discussion

Global loss of biodiversity due to climate change and land-use change continues to attract attention, but the effect of climate change and land-use on species’ range shifts remains poorly understood. The problem derives to some degree from the unrealistic land-use data and ignorance of the modulating effect of land-use change within climate scenarios on species’ range shifts. In this study, we have tried to circumvent these issues by exploring the isolated and combined climate change and land-use change effects, as well as the impact of land-use change on climate change effects, using a more realistic land-use dataset. Our analyses suggested that there is a large variability in the responses of the 23 large- and medium-sized terrestrial mammals in the giant panda range to single or combined changes in climate and land-use under different future scenarios. Moreover, our analyses showed that the modulating effects of land-use change within climate scenarios on species’ range shifts are also considerably variable. These findings should inform more useful dialogue determining the effect of climate change and land-use change on the future distribution of the 23 large- and medium-sized terrestrial mammals in the giant panda range and consequently guide future effective conservation planning.

Our projections suggested that although most of the mammalian species included in this study would experience range contractions under all dynamic climate and constant land-use scenarios, the individual species’ responses are also variable. This finding is consistent with a previous study from Zhang et al. [22], who used the climate-only models to project the future distributions of the same 23 large- and medium-sized terrestrial mammals under two emission scenarios (RCP2.6 and RCP8.5) by the 2050s and the 2070s, respectively. However, our results indicate that the proportion of species that would experience range contractions projected by the CLIM models with dynamic climate and constant land-use variables is much smaller compared to Zhang et al. [22]. This may be because the climate-only models they used generally neglected other important factors, such as land-use change, as potential drivers of species’ range shifts and therefore may have potentially overestimated the magnitude of distributional shifts of species [12,18]. Despite that, both of these studies suggested that the CO_2_ emission scenarios may play an important role in determining the magnitude of distributional shifts of species, as the average net loss of suitable habitat area across the 23 mammalian species increased with the rise in CO_2_ emission concentration. These findings indicated that the mitigation of CO_2_ emissions can help to reduce the extinction risk of species [21,27].

Our projections also suggested that both the directions and magnitudes of range shifts of the 23 mammalian species projected by the LU models with constant climate and dynamic land-use variables were quite different from the CLIM models. On the one hand, the average changes in the area of suitable habitat projected by the LU models decreased with increasing CO_2_ emissions, which was completely opposite to the trends projected by the CLIM models. Consistent with previous studies [8,9], these findings suggest that if the current climate conditions remain unchanged in the future period, future land-use change might improve the habitat quality of those mammalian species to some extent in higher CO_2_ emission scenarios, thus avoiding further loss of suitable habitat of those mammalian species in the giant panda range. However, this type of positive effect of land-use change was limited, as the magnitude of species’ range shifts projected by the CLIM models was at least two times the magnitude of species’ range shifts projected by the LU models in most cases. Previous studies have suggested that climate change can outperform land-use in determining future species distribution at a coarse scale [8,9,18]. Our findings further indicated that even at fine spatial scales (1 km × 1 km), land-use change still has a rather weak effect in determining the future range shifts of species. Despite that, it may still be too coarse to model the environmental niche of species at such spatial scales [18,28]. However, the large variation in the individual species’ responses suggest that at such spatial scales, the climate and land-use data used in this study could, to some extent, reflect the species-specific habitat relationships for those mammalian species.

Previous studies have suggested that land-use change can, to some extent, modulate the effect of climate change on species’ range shifts under all climate change scenarios [14,28]. Consistent with these previous studies, our results suggest that dynamic land-use variables can either amplify or buffer the negative effects of a changing climate, as at least 15 species would further lose additional suitable or gain less suitable habitat while the remaining species would lose less suitable habitat when both dynamic climate variables and land-use variables are considered. These findings highlight the importance of land management strategies for the conservation of mammalian species in the giant panda range, as the managers and decision-makers can implement more targeted land-use management strategies to reduce the capacity of land-use change to accelerate negative climate change impacts and/or enhance the capacity of land-use change to buffer negative climate change impacts. This type of management strategy is intuitively feasible, as land-use patterns, such as forest cover, are more locally controlled by managers and decision-makers compared to climate factors [14]. However, caution is warranted in generalizing this type of management strategy to those species that are highly spatially overlapped, as it is difficult to make effective land-use management strategies if they respond oppositely to land-use change.

Despite our study having advanced our knowledge of the effect of climate change and land-use on species’ range shifts to some extent, there are still some shortcomings in our study. First, only a global dispersal mode was considered in this study, which could lead to the underestimation of the range shifts of species. Moreover, some other abiotic and biotic variables, such as species traits (e.g., diet), range size, and population size, may also affect the response of these species to future climate change and land-use change. These variables were not incorporated into our analysis due to their unavailability. Therefore, future studies must incorporate these important variables so as to accurately predict the range shift of each species under future climate change and land-use change.

## 5. Conclusions

In conclusion, we assessed the range shifts of the 23 large- and medium-sized terrestrial mammals in the giant panda range under different future climate and land-use change scenarios based on a more realistic land-use dataset. Notably, although both the isolated climate change impacts and isolated land-use change impacts are highly variable across species, the former dominates the responses for almost all species. Moreover, the comparisons of the COMB and CLIM projections reveal that future land-use change may amplify or offset the negative effects of climate change. By explicitly incorporating informed future climate change and land-use change scenarios, we demonstrate the potential importance of relatively stable land-use patterns inside protected areas in accurately projecting future species distribution under climate change and land-use change and help identify promising land-use management strategies that can form the basis for climate change mitigation.

## Figures and Tables

**Table 1:** List of mammals, the number of grid cells where each species is present, and the training and test AUC values of the MaxEnt models for each species.

Order	Common Name	Scientific Name	Number of Records	Training AUC	Test AUC
Primates	Sichuan Snub-Nosed Monkey	*Rhinopithecus roxellana*	191	0.914 ± 0.004	0.912 ± 0.002
	Rhesus Macaque	*Macaca mulatta*	47	0.923 ± 0.008	0.917 ± 0.005
	Tibetan Macaque	*Macaca thibetana*	306	0.822 ± 0.019	0.766 ± 0.031
Carnivora	Asiatic Black Bear	*Ursus thibetanus*	537	0.909 ± 0.006	0.905 ± 0.004
	Grey Wolf	*Canis lupus*	19	0.903 ± 0.060	0.797 ± 0.075
	Red Fox	*Vulpes vulpes*	29	0.872 ± 0.010	0.862 ± 0.007
	Giant Panda	*Ailuropoda melanoleuca*	2075	0.834 ± 0.051	0.692 ± 0.085
	Chinese Red Panda	*Ailurus fulgens*	632	0.826 ± 0.015	0.824 ± 0.005
	Hog Badger	*Arctonyx albogularis*	149	0.830 ± 0.032	0.783 ± 0.014
	Masked Palm Civet	*Paguma larvata*	28	0.771 ± 0.058	0.715 ± 0.045
	Asiatic Golden Cat	*Catopuma temminckii*	23	0.891 ± 0.013	0.880 ± 0.011
	Leopard Cat	*Prionailurus bengalensis*	522	0.862 ± 0.084	0.756 ± 0.081
Artiodactyla	Forest Musk Deer	*Moschus berezovskii*	290	0.865 ± 0.014	0.841 ± 0.008
	Tufted Deer	*Elaphodus cephalophus*	786	0.827 ± 0.027	0.788 ± 0.023
	Sambar Deer	*Rusa unicolor*	271	0.898 ± 0.006	0.894 ± 0.003
	Reeves’ Muntjac	*Muntiacus reevesi*	78	0.780 ± 0.082	0.648 ± 0.105
	Takin	*Budorcas taxicolor*	1535	0.813 ± 0.014	0.796 ± 0.010
	Chinese Serow	*Capricornis sumatraensis*	914	0.918 ± 0.019	0.910 ± 0.009
	Chinese Goral	*Naemorhedus griseus*	1289	0.879 ± 0.024	0.833 ± 0.031
	Wild Boar	*Sus scrofa*	1385	0.941 ± 0.012	0.927 ± 0.009
Rodentia	Chinese Bamboo Rat	*Rhizomys sinensis*	73	0.831 ± 0.008	0.823 ± 0.007
	Malayan Porcupine	*Hystrix brachyura*	117	0.844 ± 0.015	0.836 ± 0.010
	Himalayan Marmot	*Marmota himalayana*	18	0.841 ± 0.046	0.777 ± 0.056

## Data Availability

The original contributions presented in the study are included in the article; further inquiries can be directed to the corresponding authors.

## Supplementary Materials

The following supporting information can be downloaded at: https://www.mdpi.com/article/10.3390/ani16030420/s1, Table S1: The predicted changes in the suitable habitat of the 23 large- and medium-sized terrestrial mammals in the giant panda range under different future scenarios by the CLIM models; Table S2: The predicted changes in the suitable habitat of the 23 large- and medium-sized terrestrial mammals in the giant panda range under different future scenarios by the LU models; Table S3: The predicted changes in the suitable habitat of the 23 large- and medium-sized terrestrial mammals in the giant panda range under different future scenarios by the COMB models; Figure S1: The predicted changes in the suitable habitat of the giant panda between current and different future scenarios by the CLIM models; Figure S2: The predicted changes in the suitable habitat of the giant panda between current and different future scenarios by the LU models; Figure S3: The predicted changes in the suitable habitat of the rhesus macaque between current and different future scenarios by the CLIM models; Figure S4: The predicted changes in the suitable habitat of the rhesus macaque between current and different future scenarios by the LU models.
