# Supplementary material for: The Large Variability in Response to Future Climate and Land-Use Changes Among Large- and Medium-Sized Terrestrial Mammals in the Giant Panda Range
Source: Animals (Basel). 2026 Jan 29;16(3):420. doi: 10.3390/ani16030420 (PMC12896507; doi:10.3390/ani16030420)
Supplement: Supplementary file 1 [file animals-16-00420-s001.zip › Table S1. The predicted changes in suitable habitat of the 23 large- and medium-sized terrestrial mammals in the giant panda range under di.pdf]

Table S1. The predicted changes in suitable habitat (%) of the 23 large- and medium-sized terrestrial mammals in the giant panda range under different future scenarios by CLIM models.

Speicies	Changs in suitable habitat (%)					
	2050s			2070s		
	RCP2.6	RCP4.5	RCP8.5	RCP2.6	RCP4.5	RCP8.5
Ailuropoda melanoleuca	-13.5	-19.6	-49.0	-13.8	-35.6	-54.5
Ailurus fulgens	-35.5	-46.7	-46.1	-21.2	-53.8	-72.3
Arctonyx albogularis	-29.6	-10.6	-28.1	-20.1	-32.9	-48.6
Budorcas taxicolor	-26.8	-18.5	-48.7	-23.2	-39.4	-60.1
Canis lupus	-18.1	-31.7	-87.1	-28.9	-49.6	-72.9
Capricornis sumatraensis	-26.5	-22.0	-52.0	-30.8	-35.9	-57.7
Catopuma temminckii	20.2	10.9	11.4	48.9	0.8	-5.3
Elaphodus cephalophus	6.3	-11.4	-29.3	-8.8	-9.3	-20.7
Hystrix brachyura	0.9	-5.4	0.4	10.2	-7.8	-15.3
Macaca mulatta	21.6	13.1	15.7	21.6	18.4	20.9
Macaca thibetana	1.3	-24.5	-32.4	-5.2	-17.6	-27.0
Marmota himalayana	11.9	-12.2	-37.7	11.5	-22.0	-26.5
Moschus berezovskii	7.1	-16.2	-33.2	5.4	-20.2	-32.1
Muntiacus reevesi	-10.9	-4.1	-47.3	-17.0	-21.9	-48.8
Naemorhedus griseus	-24.5	-12.6	-51.3	-21.3	-36.2	-55.0
Paguma larvata	83.2	46.1	-5.3	43.0	57.3	62.8
Prionailurus bengalensis	-22.9	-15.7	-35.2	-26.0	-28.3	-40.4
Rhinopithecus roxellana	-3.4	42.4	-43.6	-1.1	-5.0	-20.6
Rhizomys sinensis	-12.8	-7.7	-16.2	-3.7	-20.6	-39.4
Rusa unicolor	73.3	-41.5	3.8	55.0	8.0	4.6
Sus scrofa	-0.2	-8.2	-22.4	-10.5	-10.7	-19.1
Ursus thibetanus	-7.6	-15.4	-43.7	-23.8	-19.4	-37.0
Vulpes vulpes	-29.2	-7.7	26.6	7.9	-24.7	-18.2
