# Supplementary material for: The Large Variability in Response to Future Climate and Land-Use Changes Among Large- and Medium-Sized Terrestrial Mammals in the Giant Panda Range
Source: Animals (Basel). 2026 Jan 29;16(3):420. doi: 10.3390/ani16030420 (PMC12896507; doi:10.3390/ani16030420)
Supplement: Supplementary file 1 [file animals-16-00420-s001.zip › Table S2. The predicted changes in suitable habitat of the 23 large- and medium-sized terrestrial mammals in the giant panda range under di.pdf]

**Table S2.** The predicted changes in suitable habitat (%) of the 23 large- and medium-sized terrestrial mammals in the giant panda range under different future scenarios by LU models.

| Speicies | Changs in suitable habitat (%) | | | | | |
|---|---|---|---|---|---|---|
| | 2050s | | | 2070s | | |
| | RCP2.6 | RCP4.5 | RCP8.5 | RCP2.6 | RCP4.5 | RCP8.5 |
| *Ailuropoda melanoleuca* | -1.4 | -0.8 | -0.2 | -1.5 | -2.2 | -0.3 |
| *Ailurus fulgens* | -2.8 | -2.0 | -0.1 | -2.8 | -3.5 | -0.1 |
| *Arctonyx albogularis* | 2.8 | 2.0 | -0.2 | 2.7 | 5.7 | -0.3 |
| *Budorcas taxicolor* | 0.2 | 0.2 | -0.5 | 0.1 | 1.4 | -0.7 |
| *Canis lupus* | -3.1 | -3.9 | 2.8 | -2.8 | -9.1 | 3.7 |
| *Capricornis sumatraensis* | -2.8 | -2.0 | -0.9 | -2.9 | -4.6 | -1.1 |
| *Catopuma temminckii* | -7.6 | -5.5 | 1.7 | -7.4 | -7.9 | 2.3 |
| *Elaphodus cephalophus* | -2.9 | -1.7 | -0.6 | -2.9 | -4.3 | -0.7 |
| *Hystrix brachyura* | -7.4 | -5.0 | 0.4 | -7.3 | -7.8 | 0.6 |
| *Macaca mulatta* | -6.8 | -4.9 | 0.0 | -6.8 | -7.2 | 0.0 |
| *Macaca thibetana* | -3.3 | -1.8 | -0.4 | -3.4 | -3.3 | -0.5 |
| *Marmota himalayana* | -4.2 | -3.8 | 3.4 | -3.8 | -4.8 | 4.4 |
| *Moschus berezovskii* | -2.1 | -1.3 | -0.4 | -2.1 | -4.0 | -0.4 |
| *Muntiacus reevesi* | -4.5 | -3.1 | 0.0 | -4.5 | -5.2 | 0.0 |
| *Naemorhedus griseus* | -1.4 | -1.0 | -0.7 | -1.5 | -3.2 | -0.9 |
| *Paguma larvata* | -9.2 | -7.8 | 3.7 | -8.8 | -13.6 | 4.8 |
| *Prionailurus bengalensis* | -3.0 | -1.8 | -0.3 | -3.0 | -4.0 | -0.3 |
| *Rhinopithecus roxellana* | -1.8 | -1.6 | 0.1 | -1.8 | -2.2 | 0.2 |
| *Rhizomys sinensis* | -4.7 | -3.4 | -0.1 | -4.7 | -5.8 | -0.1 |
| *Rusa unicolor* | -0.9 | -0.4 | -0.5 | -1.2 | 2.0 | -0.6 |
| *Sus scrofa* | -4.7 | -3.1 | 0.0 | -4.7 | -5.2 | 0.0 |
| *Ursus thibetanus* | -2.6 | -1.6 | -0.4 | -2.7 | -3.5 | -0.5 |
| *Vulpes vulpes* | -18.2 | -14.7 | 3.1 | -17.6 | -20.9 | 4.8 |
