# Supplementary material for: The Large Variability in Response to Future Climate and Land-Use Changes Among Large- and Medium-Sized Terrestrial Mammals in the Giant Panda Range
Source: Animals (Basel). 2026 Jan 29;16(3):420. doi: 10.3390/ani16030420 (PMC12896507; doi:10.3390/ani16030420)
Supplement: Supplementary file 1 [file animals-16-00420-s001.zip › Table S3. The predicted changes in suitable habitat of the 23 large- and medium-sized terrestrial mammals in the giant panda range under di.pdf]

Table S3. The predicted changes in suitable habitat (%) of the 23 large- and medium-sized terrestrial mammals in the giant panda range under different future scenarios by COMB models.

Speicies	Changs in suitable habitat (%)					
	2050s			2070s		
	RCP2.6	RCP4.5	RCP8.5	RCP2.6	RCP4.5	RCP8.5
Ailuropoda melanoleuca	-14.4	-20.3	-49.2	-14.6	-37.4	-54.8
Ailurus fulgens	-37.2	-47.9	-46.2	-23.4	-56.1	-72.4
Arctonyx albogularis	-26.9	-8.5	-28.4	-17.7	-25.9	-49.1
Budorcas taxicolor	-26.5	-18.3	-49.1	-22.7	-39.3	-60.5
Canis lupus	-20.1	-33.3	-86.7	-29.8	-52.4	-71.8
Capricornis sumatraensis	-28.3	-23.4	-52.7	-32.6	-38.7	-58.6
Catopuma temminckii	10.9	4.8	13.4	36.9	-6.1	-3.6
Elaphodus cephalophus	4.5	-12.4	-30.1	-10.1	-12.1	-22.1
Hystrix brachyura	-6.6	-10.2	0.8	2.2	-15.2	-14.8
Macaca mulatta	12.1	7.8	15.9	12.8	9.0	21.3
Macaca thibetana	-1.0	-25.4	-32.7	-7.2	-19.7	-27.5
Marmota himalayana	7.4	-17.6	-33.8	7.4	-30.3	-21.5
Moschus berezovskii	5.1	-17.3	-33.9	3.3	-24.1	-33.0
Muntiacus reevesi	-14.6	-6.8	-47.4	-20.2	-25.7	-48.9
Naemorhedus griseus	-25.5	-13.3	-51.9	-22.2	-38.5	-55.7
Paguma larvata	67.7	36.0	-1.9	31.3	38.8	68.1
Prionailurus bengalensis	-24.7	-17.2	-35.6	-27.9	-31.6	-40.9
Rhinopithecus roxellana	-5.5	39.1	-43.6	-3.2	-8.1	-20.3
Rhizomys sinensis	-16.8	-10.5	-16.5	-8.1	-24.7	-39.7
Rusa unicolor	68.2	-42.1	3.4	53.2	7.1	3.9
Sus scrofa	-4.3	-10.7	-22.6	-13.9	-15.8	-19.7
Ursus thibetanus	-9.2	-16.3	-44.0	-25.1	-22.1	-37.7
Vulpes vulpes	-42.0	-21.6	30.1	-9.5	-40.6	-14.8
