# Supplementary figures and images for: The Large Variability in Response to Future Climate and Land-Use Changes Among Large- and Medium-Sized Terrestrial Mammals in the Giant Panda Range
Source: Animals (Basel). 2026 Jan 29;16(3):420. doi: 10.3390/ani16030420 (PMC12896507; doi:10.3390/ani16030420)

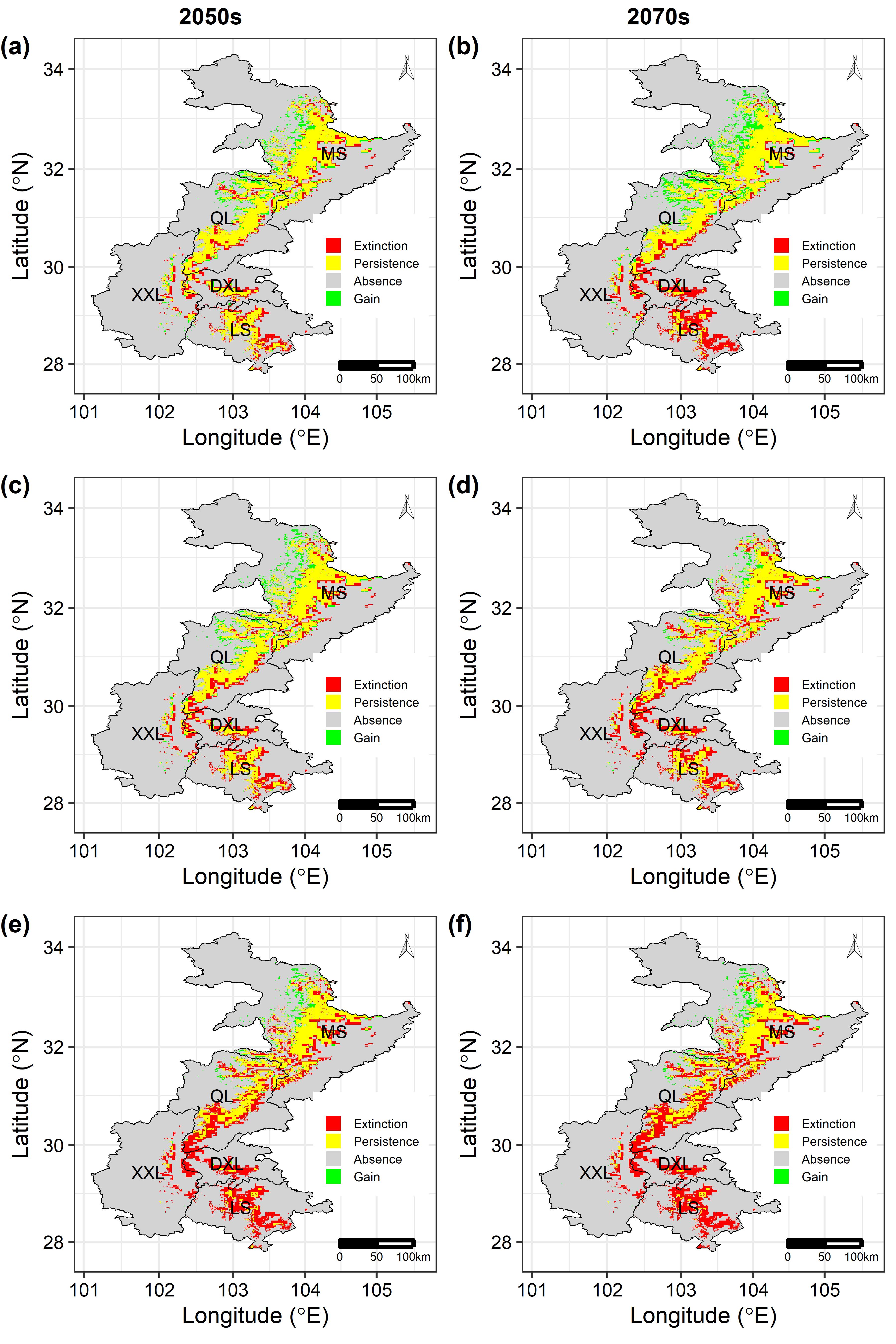

Supplement: Supplementary file 1 [file animals-16-00420-s001.zip › Figure S1. The predicted changes in suitable habitat of the giant panda between current and different future scenarios by the CLIM models..jpg]

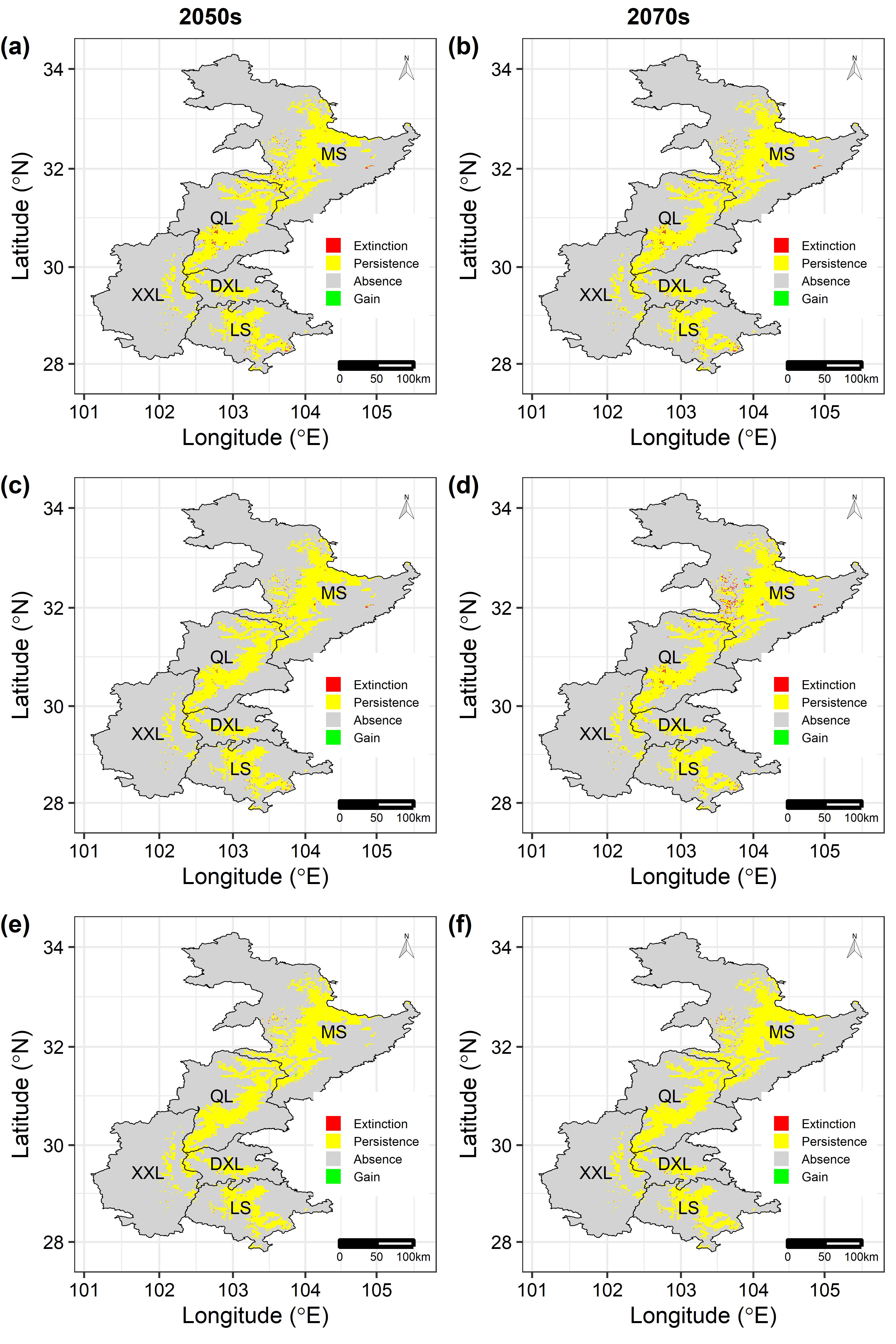

Supplement: Supplementary file 1 [file animals-16-00420-s001.zip › Figure S2. The predicted changes in suitable habitat of the giant panda between current and different future scenarios by the LU models..jpg]

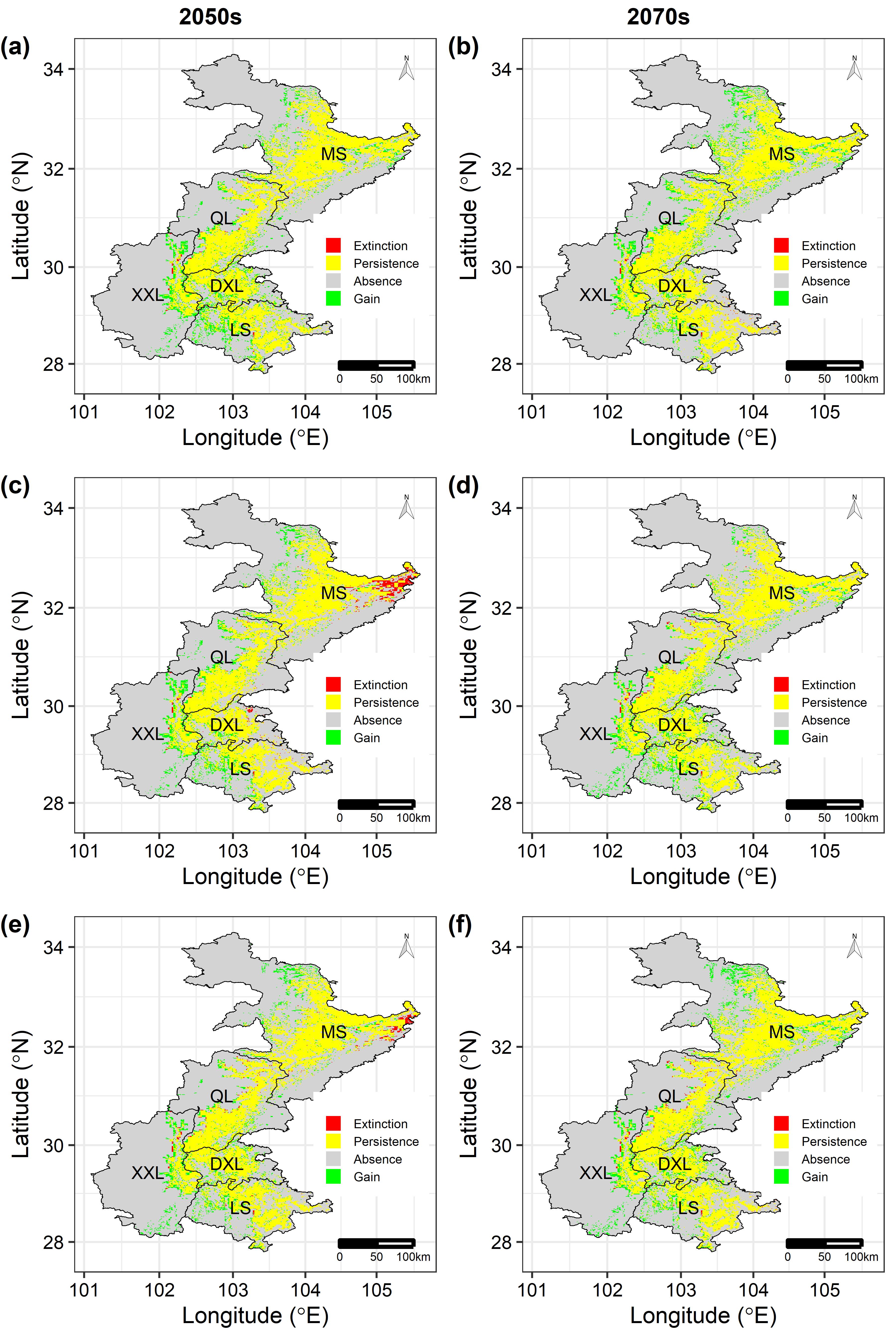

Supplement: Supplementary file 1 [file animals-16-00420-s001.zip › Figure S3. The predicted changes in suitable habitat of the Rhesus Macaque between current and different future scenarios by the CLIM models..jpg]

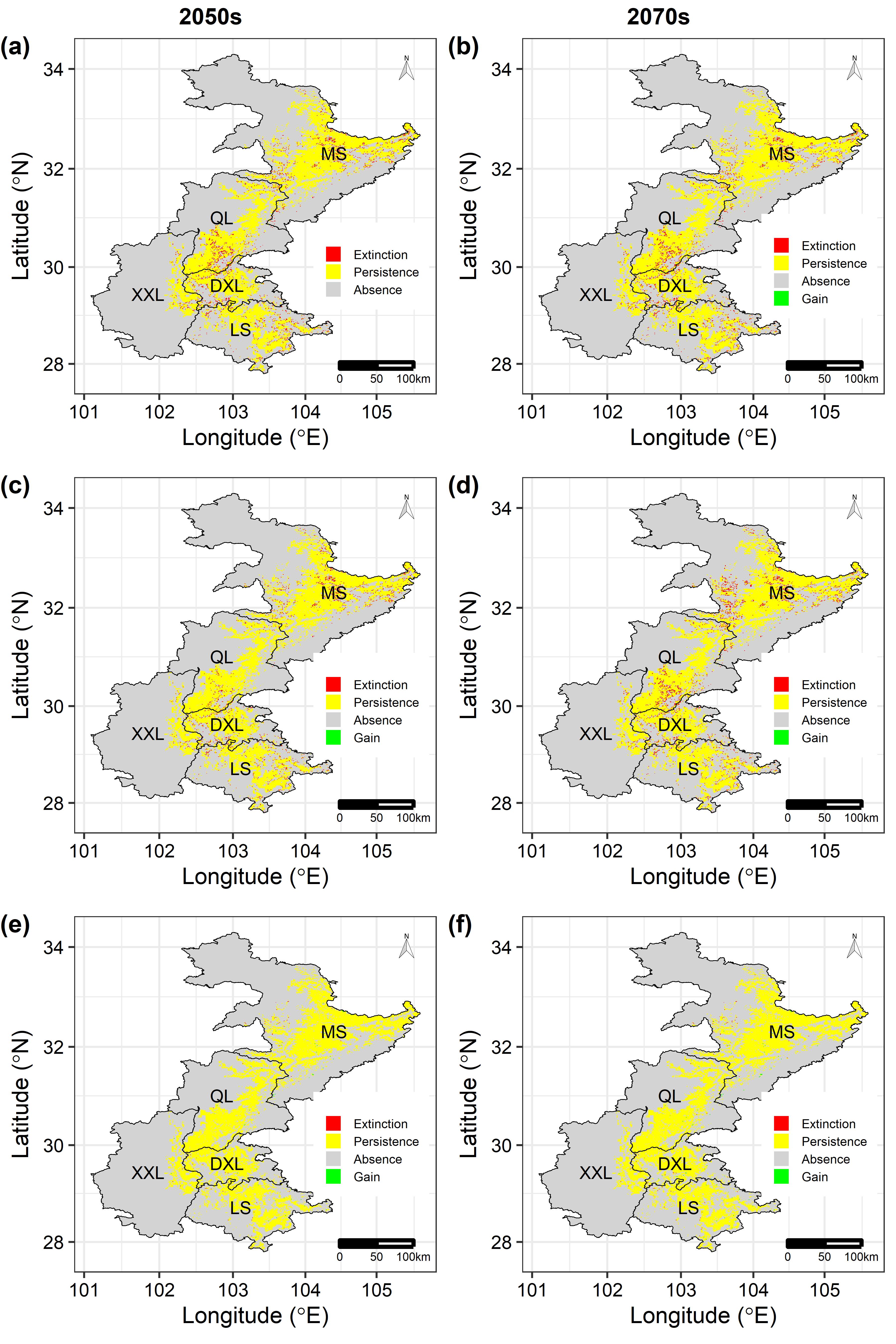

Supplement: Supplementary file 1 [file animals-16-00420-s001.zip › Figure S4. The predicted changes in suitable habitat of the Rhesus Macaque between current and different future scenarios by the LU models..jpg]
